# Multimodal Optical and Ratiometric ATR-FTIR Discrimination of Mixed Aerosol Components Using pH-Responsive Methylcellulose–Phenol Red Films

**DOI:** 10.3390/s26123839

**Published:** 2026-06-17

**Authors:** Chinmaya Mutalik, Rachel Redmann, Sarah Bose, Bryan Tassin, Amy Phou, Chad J. Roy

**Affiliations:** Center for Airborne Infection & Transmission Science, Tulane University School of Medicine, New Orleans, LA 70112, USA; cmutalik@tulane.edu (C.M.); rredmann@tulane.edu (R.R.); aphou@tulane.edu (A.P.)

**Keywords:** methylcellulose–phenol red films, ATR-FTIR spectroscopy, ratiometric sensing, bioaerosol detection, pH-responsive sensing

## Abstract

Breath aerosol analysis requires low-cost sensing substrates capable of capturing aerosolized biomolecular components while preserving chemically interpretable readouts. Here, methylcellulose–phenol red (MCPR) films are evaluated as multimodal sensing substrates using model bioaerosols consisting of sodium sulfate, bovine serum albumin (BSA), and polystyrene latex particles under acidic, neutral, and alkaline pH conditions. ATR-FTIR spectroscopy revealed inverse pH-dependent trends in sulfate (1000–1100 cm^−1^) and protein amide (1500–1700 cm^−1^) spectral regions. A sulfate-to-protein AUC ratio increased from 0.86 ± 0.01 at pH 4 to 3.56 ± 0.32 at pH 10, demonstrating ratiometric compositional discrimination of ionic and proteinaceous aerosol fractions. UV–Vis spectroscopy showed pH-dependent λmax shifts from 432 to 556 nm, confirming the preservation of phenol red optical responsiveness after aerosol exposure. FTIR-derived ratio metrics correlated linearly with optical responses, indicating coupled vibrational and optical sensing behavior. SEM-EDS analysis of methylcellulose capture films confirmed deposition of sulfate, proteinaceous, and particulate aerosol components, supporting the platform’s suitability for multimodal spectroscopic sensing. These findings establish MCPR films as integrated capture-and-sensing substrates capable of coupling optical pH responsiveness with label-free vibrational analysis, supporting future development of low-cost breath-relevant aerosol sensing platforms.

## 1. Introduction

Breath-based diagnostics have emerged as an attractive alternative to invasive clinical sampling approaches because exhaled aerosols contain molecular signatures that reflect physiological and pathological processes within the respiratory microenvironment [1,2,3,4,5]. Unlike blood or sputum collection, breath sampling is non-invasive, repeatable, and well-suited for point-of-care screening applications [6,7,8]. Infrared spectroscopy has increasingly been explored as a label-free technique for analyzing breath-derived biomarkers due to its ability to provide direct chemical fingerprints without complex sample preparation. Among candidate sensing materials for breath analysis, polymer films functionalized with pH-responsive dyes offer a compelling approach because they combine analyte capture with visual and spectroscopic reporting capabilities [9,10,11,12,13]. Methylcellulose (MC) is a hydrophilic polysaccharide widely used in biomedical and sensing applications owing to its film-forming ability, chemical stability, and compatibility with aqueous environments [14,15,16].

Incorporation of phenol red into methylcellulose matrices produces films that undergo reversible optical transitions from yellow to red across physiologically relevant pH ranges, enabling both qualitative visual detection and quantitative spectroscopic analysis. Despite these advantages, the performance of methylcellulose–phenol red (MCPR) films for aerosol capture and compositional analysis has not been systematically investigated using multimodal spectroscopic approaches. Previous polymer-based sensing films, such as cellulose-derived, hydrogel-based, and dye-immobilized substrates, have been investigated for pH monitoring, environmental sensing, food packaging, and biomedical purposes. These devices often offer colorimetric or fluorescence-based readouts and are useful for quick visual or optical detection [14,15,16,17,18,19,20]. However, most reported pH-responsive films do not allow label-free vibrational discrimination between chemically different aerosol components. Separately, FTIR-based aerosol characterization techniques can detect chemical signatures of biological and environmental particles, but they are rarely coupled with responsive polymer capture substrates. The MCPR platform used in this study fills this gap by combining methylcellulose-based aerosol capture, phenol red-mediated optical responsiveness, and ratiometric ATR-FTIR discrimination of ionic and proteinaceous aerosol fractions within a single sensing interface [21,22,23].

In particular, the relationship between optical colorimetric response and vibrational spectral signatures following aerosol deposition remains poorly understood. Attenuated total reflectance Fourier-transform infrared (ATR-FTIR) spectroscopy provides a powerful platform for analyzing surface-deposited biomolecules because it enables direct measurement of molecular vibrational fingerprints from solid substrates with minimal preparation. The technique has been widely used for analyzing biological samples, polymers, and environmental particulates, including aerosols and microbial deposits [24,25,26,27]. However, integration of ATR-FTIR with optical indicator-based polymer sensing platforms for aerosol characterization has not been previously explored.

In this work, we present a multimodal spectroscopic validation of MCPR films as aerosol-responsive sensing substrates by combining ATR-FTIR spectroscopy, UV–Vis absorbance measurements, scanning electron microscopy (SEM), and energy-dispersive X-ray spectroscopy (EDS). Using model aerosols representing ionic and proteinaceous breath components, we demonstrate that MCPR films simultaneously capture aerosolized species and generate interpretable chemical signatures across multiple sensing modalities. A ratiometric FTIR analysis strategy based on sulfate-to-protein spectral integration further enables quantitative compositional discrimination across physiologically relevant pH conditions [28]. These results establish MCPR films as promising candidates for future breath-based diagnostic sensing platforms.

## 2. Materials and Methods

### 2.1. Preparation of MC and MCPR Films

Methylcellulose (4 wt%; Thermo Scientific, Waltham, MA, USA) was dissolved in deionized water at 60 °C and stirred continuously until a homogenous solution was produced. Phenol red (0.02 wt%; Sigma-Aldrich, St. Louis, MO, USA) was added as a pH indicator and properly mixed before casting. The resulting MCPR solution was cast into polystyrene Petri dishes and dried in a vacuum oven at 40 °C overnight to produce continuous, handleable films. This gentle drying temperature was chosen to promote progressive water removal while reducing thermal stress, film cracking, and phenol red breakdown or redistribution within the methylcellulose matrix. To assess aerosol capture independent of optical indicator response, control methylcellulose films containing no phenol red were made using the same technique. The film thickness was not systematically tuned in by the current proof-of-concept investigation; future research will include controlled casting volumes, profilometry or micrometer-based thickness measurements, and evaluation of thickness-dependent optical and ATR-FTIR responses [29].

### 2.2. Generation and Collection of Model Bioaerosols

Model aerosol suspensions were prepared in a final volume of 10 mL containing sodium sulfate and BSA at final concentrations of 100 µg/mL each, corresponding to 1 mg of each component per preparation. PSL microspheres (1.04 µm mean diameter, 1% solids; Bangs Laboratories, Fishers, IN, USA) were added by diluting 500 µL of stock suspension into the final 10 mL formulation, corresponding to an estimated ~8.1 × 10^9^ PSL particles per preparation, or ~8.1 × 10^8^ particles/mL. Sodium sulfate, BSA, and PSL particles were chosen as representatives of chemically distinct fractions of interest in biological and breath-related aerosol systems. Deposition morphology and capture behavior were evaluated under control conditions with sodium sulfate as an inorganic ionic surrogate, BSA as a representative proteinaceous biomolecular component, and PSL particles as inert particulate aerosol surrogates. The model system allowed the ionic, protein-associated, and particulate contributions to be evaluated separately before future studies using real exhaled breath aerosols. Aerosols were generated using a three-jet Collison nebulizer operated under controlled airflow conditions and introduced into a controlled impinger-based aerosol collection system (Biaera Technologies, Hagerstown, MD, USA) [5,30,31]. MCPR films were positioned within the collection chamber to maximize particle deposition efficiency. The pH of the aerosol collection medium was adjusted to pH 4, pH 7, and pH 10 using dilute hydrochloric acid or sodium hydroxide to evaluate pH-dependent sensor responses across physiologically relevant conditions. The selected pH values are representative of controlled acidic, neutral, and alkaline model conditions to evaluate the dynamic range of the MCPR sensing matrix. These values were not meant as direct diagnostic thresholds of specific respiratory diseases but represent analytically useful microenvironmental conditions for assessing pH-dependent optical and vibrational sensing behavior.

### 2.3. ATR-FTIR Spectroscopic Analysis

An ATR-FTIR (Lyza 7000, Anton Paar, Ashland, VA, USA) spectrophotometer and a diamond-crystal ATR accessory (IRIS, Pike Technologies, Madison, WI, USA) were utilized to record over the spectral range 600–2000 cm^−1^. ATR-FTIR spectra were exported as absorbance versus wavenumber and processed using OriginPro 8 and GraphPad Prism version 11.0. Prior to quantitative analysis, spectra were corrected by subtraction of the corresponding blank methylcellulose control spectrum to remove background contributions from the polymer substrate and ATR crystal. Local linear baseline correction was then applied within predefined spectral windows corresponding to sulfate stretching vibrations (1000–1100 cm^−1^) and protein amide vibrations (1500–1700 cm^−1^). Area under the curve (AUC) values were calculated from the baseline-corrected absorbance spectra using trapezoidal numerical integration within these fixed spectral limits. Integration boundaries were applied consistently across all samples to ensure reproducible comparison between experimental conditions. A sulfate-to-protein ratio metric was subsequently calculated from integrated absorbance values obtained from these two spectral windows to provide a normalized compositional indicator independent of absolute signal intensity differences between samples. Integration values were obtained from three independent experimental replicates per condition and are reported as mean ± standard deviation. No global spectral normalization was applied prior to integration; instead, blank subtraction and local baseline correction were used to preserve relative absorbance differences between sulfate- and protein-associated spectral regions. Second-derivative spectral transformation and Gaussian peak fitting were additionally performed to improve the resolution of overlapping vibrational features and support peak assignment; however, quantitative AUC measurements were obtained from baseline-corrected absorbance spectra rather than derivative spectra. Statistical analyses and data visualization were carried out using GraphPad Prism and Origin Pro 8.

### 2.4. UV–Vis Spectroscopy

UV–Vis absorbance spectra were collected between 400 and 650 nm to characterize pH-dependent optical transitions associated with phenol red using a Genesys 140 UV-Vis Spectrophotometer (Thermo Scientific, Waltham, MA, USA). The wavelength corresponding to maximum absorbance (λmax) was determined for each condition and used as an optical indicator of film response. Changes in λmax were correlated with FTIR-derived spectral ratio metrics to evaluate coupled optical and vibrational sensing behavior. Statistical analyses and data visualization were carried out using GraphPad Prism and Origin Pro 8.

### 2.5. Scanning Electron Microscopy and Energy-Dispersive X-Ray Spectroscopy

Surface morphology and aerosol deposition patterns on methylcellulose (MC) films were examined using a scanning electron microscope (Hitachi SU 3500, Tokyo, Japan). Elemental composition of deposited particles was analyzed using energy-dispersive X-ray spectroscopy to confirm the presence of sulfate-derived sulfur signals and carbon-rich proteinaceous material following aerosol capture.

## 3. Results and Discussion

### 3.1. Morphological and Elemental Validation of Aerosol Capture on Methylcellulose Films

**Figure 1:**
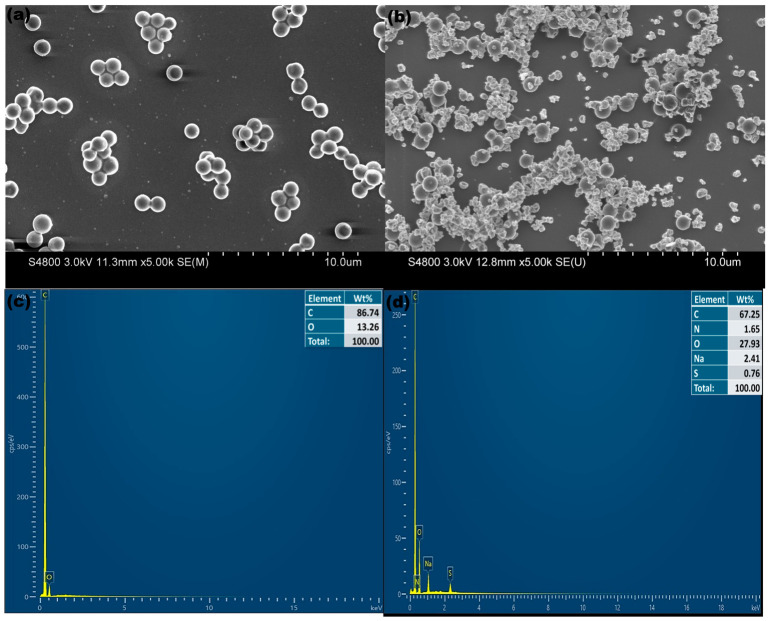
**Morphological and compositional validation of aerosol deposition onto MC films using SEM and EDS analysis:** (**a**) Scanning electron micrograph showing spherical polystyrene latex (PSL) particles deposited on the MC film surface, confirming successful capture of model aerosol particles with preserved morphology. (**b**) SEM image following mixed aerosol exposure (BSA + sulfate + PSL beads) demonstrating heterogeneous surface coverage consistent with co-deposition of ionic and proteinaceous aerosol components. (**c**) Energy-dispersive X-ray spectroscopy (EDS) spectrum of the untreated methylcellulose control film showing dominant carbon and oxygen signals characteristic of the cellulose polymer matrix. (**d**) EDS spectrum of aerosol-exposed MC film confirming additional elemental contributions, including nitrogen, sodium, and sulfur, consistent with deposition of protein and sulfate-containing aerosol species. Scale bars represent 10 μm.

Film formation was confirmed by the production of continuous, handleable dried polymer substrates suitable for aerosol exposure and spectroscopic analysis. SEM imaging further confirmed preservation of surface integrity after aerosol deposition. Scanning electron microscopy (SEM) confirmed consistent deposition of model aerosol components onto methylcellulose (MC) film surfaces following impinger-assisted aerosol collection (Figure 1). Discrete spherical particles consistent with polystyrene latex (PSL) aerosol surrogates were clearly visible across MC film surfaces after PSL-only exposure (Figure 1a) [32]. In contrast, films exposed to mixed aerosols containing PSL particles, sodium sulfate, and bovine serum albumin (BSA) exhibited heterogeneous particulate aggregates distributed across the polymer surface (Figure 1b), consistent with deposition of chemically diverse aerosol constituents [33]. Importantly, the methylcellulose substrate retained structural integrity throughout aerosol exposure and post-deposition drying, with no evidence of cracking or surface deformation. This observation supports the suitability of MC films as stable passive capture substrates for subsequent spectroscopic analysis.

Energy-dispersive X-ray spectroscopy (EDS) further confirmed the successful deposition of inorganic aerosol components. Control MC films showed dominant carbon and oxygen peaks consistent with the cellulose-based polymer matrix (Figure 1c). In contrast, aerosol-exposed MC films exhibited additional sodium and sulfur peaks (Figure 1d), confirming the presence of deposited sodium sulfate particles. Increased carbon-rich surface contributions were also consistent with adsorption of PSL particles and BSA protein components [15]. Together, these results demonstrate that methylcellulose films function as effective aerosol capture substrates capable of retaining mixed particulate, ionic, and proteinaceous components. Incorporation of phenol red into this capture matrix therefore enables the development of MCPR films that combine efficient aerosol collection with multimodal optical and vibrational sensing functionality. Because aerosol capture occurs at the methylcellulose matrix level, subsequent incorporation of phenol red enables the resulting MCPR films to function as integrated capture-and-sensing substrates without altering deposition behavior [34].

### 3.2. Optical Response of MCPR Films Across Physiologically Relevant pH Conditions

**Figure 2:**
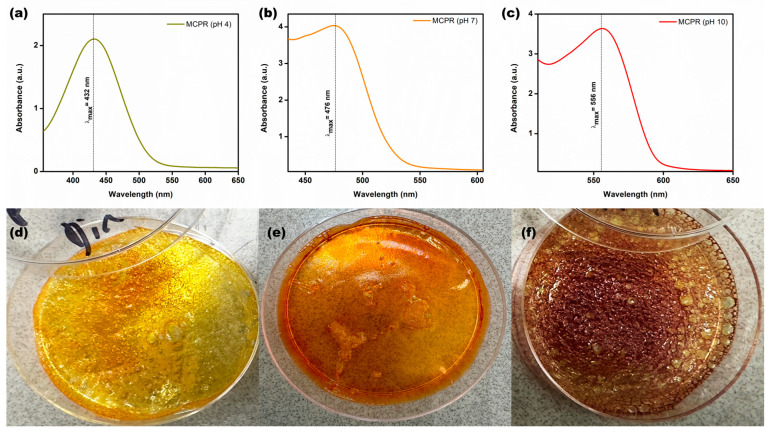
**Optical response of MCPR films across physiologically relevant pH conditions following exposure to mixed model aerosols: (a–c) UV-Vis absorbance spectra of MCPR films exposed to mixed model aerosols composed of sodium sulfate (inorganic salt component), bovine serum albumin (protein component), and polystyrene latex (PSL) beads (particulate surrogate component) at pH 4, 7, and 10.** The spectra show a progressive redshift of the absorbance maximum (λmax) from 432 nm to 556 nm, consistent with protonation-dependent transitions of phenol red embedded within the methylcellulose matrix. (**d**–**f**) Representative photographs of MCPR films corresponding to pH 4, 7, and 10 conditions demonstrating visually distinguishable colorimetric transitions from yellow to orange and red, confirming preservation of indicator responsiveness following aerosol deposition.

MCPR films exhibited clear and reproducible pH-dependent optical transitions consistent with the indicator behavior of phenol red incorporated within the methylcellulose matrix (Figure 2) [35]. UV-Vis absorbance spectra showed a progressive redshift in the absorbance maximum (λmax) from approximately 432 nm at pH 4 to 476 nm at pH 7 and 556 nm at pH 10 (Figure 2a–c). These shifts correspond to well-established protonation-deprotonation equilibria of phenol red and confirm that the dye retained its expected responsiveness after incorporation into the polymer sensing film [36]. Importantly, comparable spectral transitions were observed after aerosol exposure of the films, demonstrating that the indicator retained its responsiveness and remained spectroscopically accessible within the MCPR matrix following deposition of mixed aerosol components [37].

Representative photographs of the films further confirmed visually distinguishable color transitions from yellow (acidic) to orange (neutral) and red (alkaline) conditions (Figure 2d–f), supporting the spectroscopic observations and highlighting the potential of MCPR films for rapid visual screening applications. Similar phenol red-based optical transitions have been widely used in polymer-supported pH sensing systems due to their stability, reversibility, and compatibility with aqueous biological environments [38]. Together, these results demonstrate that MCPR films function as stable pH-responsive optical substrates capable of maintaining indicator performance after aerosol deposition, supporting their use as dual-mode platforms for combined optical and vibrational analysis of breath-relevant aerosol samples. The agreement between spectroscopic λmax shifts and visible colorimetric transitions further confirms that MCPR films preserve indicator accessibility within the polymer network after aerosol deposition [19].

### 3.3. ATR-FTIR Discrimination of Sulfate and Protein Contributions in Mixed Aerosol Deposits

**Figure 3:**
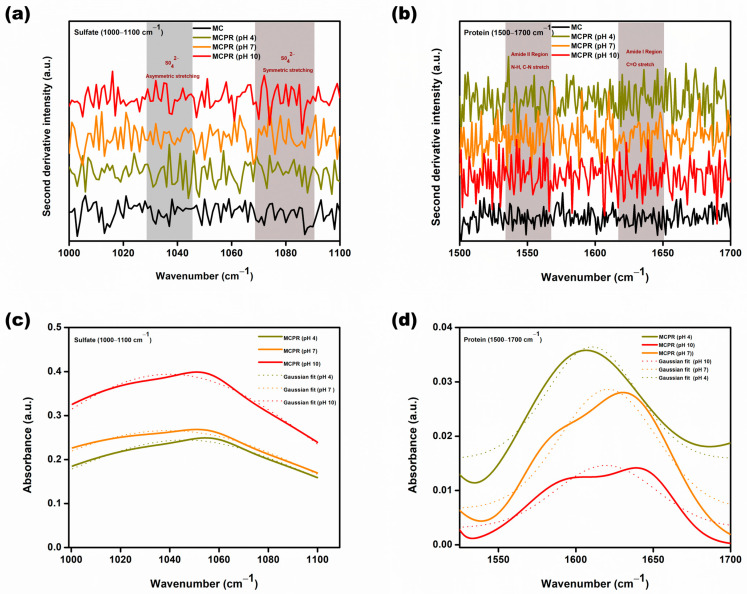
**ATR-FTIR discrimination of sulfate and protein vibrational signatures across pH conditions using second-derivative spectroscopy and Gaussian peak fitting:** (**a**) Second-derivative ATR-FTIR spectra highlighting sulfate vibrational features within the 1000–1100 cm^−1^ region for MCPR films exposed to mixed aerosols across pH conditions, with shaded regions indicating asymmetric and symmetric SO_4_^2−^ stretching bands. (**b**) Second-derivative ATR-FTIR spectra showing protein-associated vibrational features within the 1500–1700 cm^−1^ region corresponding to amide I and amide II band contributions. (**c**) Gaussian peak fitting of the sulfate spectral region demonstrating progressive enhancement of sulfate-associated absorbance with increasing pH. (**d**) Gaussian peak fitting of the protein amide region showing decreasing protein-associated absorbance intensity across increasing pH conditions.

ATR-FTIR measurements revealed clear and reproducible differences between sulfate-associated and protein-associated vibrational features across the investigated pH range (Figure 3). Reference ATR-FTIR spectra of unexposed MC control films were included in Supplementary Figure S1 to show the baseline polymer contribution before aerosol exposure. Application of second-derivative spectral processing improved resolution within the sulfate stretching region (1000–1100 cm^−1^), allowing subtle variations in overlapping vibrational contributions to be distinguished more clearly. These derivative spectra showed a progressive strengthening of sulfate-related features as the environment shifted from acidic to alkaline conditions (Figure 3a), consistent with enhanced detectability of inorganic sulfate species within the MCPR sensing matrix. Similar sulfate band assignments within this spectral window are widely reported in vibrational analyses of environmental and biological aerosol components. In contrast, second-derivative analysis of the protein-associated amide region (1500–1700 cm^−1^) showed a gradual reduction in band intensity with increasing pH (Figure 3b). This trend is consistent with pH-dependent changes in protein adsorption behavior and interactions with hydrophilic polymer matrices, which can influence the apparent intensity of amide I and amide II vibrational modes in surface-sensitive ATR measurements [26,39,40,41].

To further evaluate these compositional trends, Gaussian peak fitting was applied to both spectral regions. Fitted profiles within the sulfate stretching window confirmed a systematic increase in sulfate-associated absorbance contributions from acidic to alkaline conditions (Figure 3c). Conversely, Gaussian fitting of the amide spectral region demonstrated decreasing protein-associated absorbance intensity across the same pH range (Figure 3d), supporting the presence of opposing spectral responses between ionic and proteinaceous aerosol components deposited within the MCPR films. Taken together, these complementary spectral behaviors demonstrate that combining second-derivative ATR-FTIR processing with Gaussian peak fitting enables reliable discrimination between sulfate and protein contributions within mixed aerosol deposits. Importantly, this capability allows chemically distinct aerosol components to be resolved within a single label-free sensing platform, supporting the feasibility of MCPR films as multimodal substrates for compositional breath-relevant aerosol analysis [42,43].

### 3.4. Ratiometric FTIR Analysis Enables Compositional Discrimination

**Figure 4:**
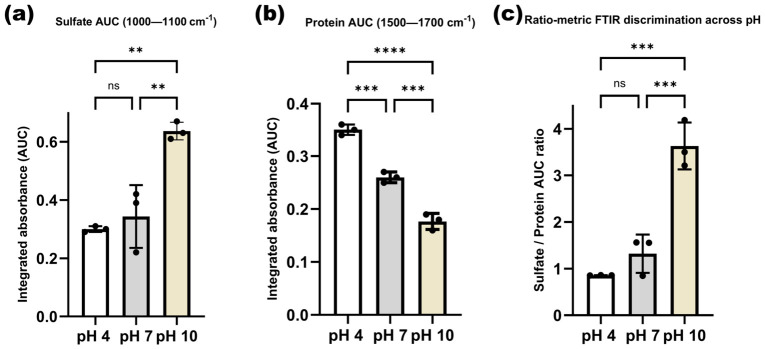
**Quantitative ATR-FTIR discrimination of sulfate and protein spectral signatures across pH conditions:** (**a**) Integrated absorbance (AUC) within the sulfate vibrational region (1000–1100 cm^−1^) showing increasing sulfate contribution with increasing pH. (**b**) Integrated absorbance (AUC) within the protein amide region (1500–1700 cm^−1^) showing decreasing protein-associated signal intensity with increasing pH. (**c**) Sulfate-to-protein AUC ratio demonstrating enhanced ratiometric compositional discrimination across conditions. Bars represent mean ± standard deviation (*n* = 3). Statistical significance was evaluated using one-way ANOVA followed by Tukey’s post hoc multiple-comparison test. Significance denoted at ** *p* > 0.001, *** *p* > 0.0001, **** *p* > 0.00001.

Quantitative integration of ATR-FTIR spectral regions corresponding to sulfate and protein vibrational modes revealed clear and opposing compositional trends across the investigated pH range (Figure 4). Area under the curve (AUC) measurements obtained from baseline-corrected ATR-FTIR spectra within the sulfate stretching window (1000–1100 cm^−1^) increased progressively from acidic to alkaline conditions (Figure 4a), indicating enhanced sulfate-associated vibrational contributions under higher pH environments. The higher variability in sulfate AUC at pH 7 is most likely due to changes in local aerosol deposition, film hydration, and ATR contact under an intermediate pH environment. Because pH 7 represents a transition state between acidic and alkaline film environments, minor variations in aerosol loading or film-ATR interaction may have a greater effect on sulfate-associated signal intensity. Individual replicate results were kept in Figure 4 to highlight this variability. In contrast, integrated absorbance within the protein amide region (1500–1700 cm^−1^) showed a systematic decrease across the same pH range (Figure 4b), consistent with reduced protein-associated signal contributions resulting from changes in adsorption behavior at the polymer interface. Similar sulfate and amide spectral assignments are well established in vibrational spectroscopy analyses of environmental aerosols and biological macromolecules [39,41,44]. These opposing spectral responses enabled the construction of a sulfate-to-protein AUC ratio as a normalized compositional indicator independent of absolute signal intensity variation. The resulting ratio increased from 0.86 ± 0.01 at pH 4 to 3.56 ± 0.32 at pH 10 (Figure 4c), corresponding to more than a four-fold increase across the investigated pH range and demonstrating strong sensitivity of the MCPR sensing platform to relative changes in ionic and proteinaceous aerosol contributions. The opposite trends for sulfate- and protein-associated signals likely reflect pH-dependent changes in the local microenvironment of the MCPR film. Methylcellulose provides a hydrophilic matrix rich in hydroxyl groups, which can retain deposited aerosol components through wetting, hydrogen bonding, and physical adsorption. Phenol red undergoes protonation–deprotonation transitions within the pH range studied, which may affect the local charge distribution and hydration within the polymer network. The higher AUC values in the sulfate region under alkaline conditions may be due to increased deprotonation in the film, which may improve the relative retention or detection of the ionic species associated with sulfate. BSA adsorption and amide-band intensity, on the other hand, depend on protein charge state, hydration, and protein–polymer interactions, which may reduce the apparent amide contribution at elevated pH. The coupled effects may provide a potential cause for the observed increase in the sulfate-associated signal and decrease in the protein-associated signal across the pH range.

Ratiometric sensing approaches are widely used in optical and vibrational sensor systems because they improve analytical robustness by minimizing baseline drift and environmental variability effects. The monotonic increase observed in the sulfate-to-protein ratio therefore provides strong evidence that MCPR films support reliable compositional discrimination between chemically distinct aerosol fractions using label-free ATR-FTIR measurements [45,46,47]. Importantly, the divergence between sulfate- and protein-associated spectral contributions suggests that local protonation equilibria within the phenol red-modified methylcellulose matrix influence ionic adsorption behavior while simultaneously modulating protein–film interactions. This coupled response enables ratiometric discrimination of mixed aerosol composition within a single sensing interface and provides a mechanistic basis for extending this approach toward breath-relevant aerosol analysis. Together, these results demonstrate that sulfate-to-protein ATR-FTIR ratio metrics provide a chemically interpretable sensing parameter for resolving mixed aerosol composition within pH-responsive polymer capture substrates [5,27,28].

### 3.5. Correlation Between Optical and Vibrational Sensing Responses in MCPR Films

**Figure 5:**
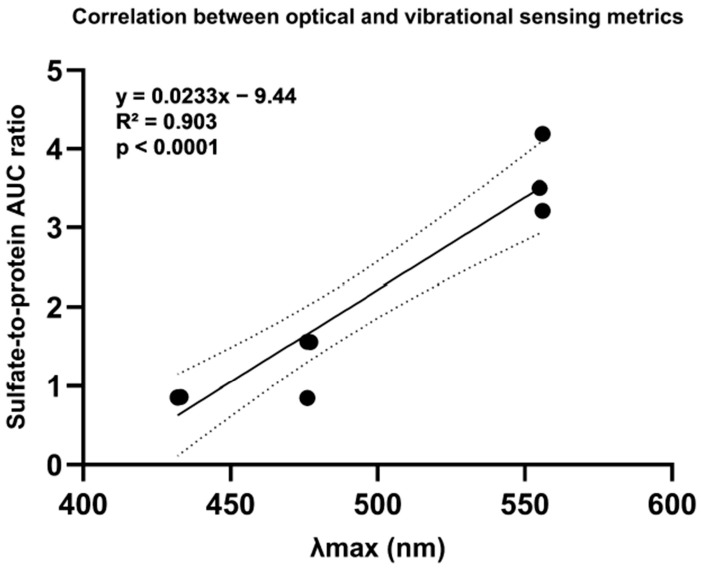
**Correlation between optical and vibrational sensing responses of MCPR films.** Linear regression analysis showing the relationship between UV–Vis absorbance maximum (λmax) and the sulfate-to-protein ATR-FTIR AUC ratio across pH conditions. Each point represents an independent experimental replication (*n* = 3 per condition). The solid line represents the best-fit regression, and dotted lines indicate the 95% confidence interval of the regression model.

A strong positive relationship was observed between UV–Vis absorbance maxima (λmax) and ATR-FTIR-derived sulfate-to-protein AUC ratios across the investigated pH range (Figure 5). Linear regression analysis demonstrated a high degree of agreement between the two sensing metrics (R^2^ = 0.903, *p* < 0.0001), indicating that changes in optical colorimetric response closely tracked compositional variations detected through vibrational spectroscopy. Because the λmax shift of phenol red reflects protonation–deprotonation equilibria within the methylcellulose sensing matrix, the observed correlation suggests that local pH-dependent chemical environments influencing dye behavior simultaneously modulate adsorption and spectral contributions of sulfate and protein aerosol components at the film surface. This relationship supports the interpretation that optical and vibrational responses arise from a shared underlying chemical microenvironment within the MCPR matrix rather than independent sensing processes. The strong linear relationship observed between λmax shifts and sulfate-to-protein spectral ratios further demonstrates that rapid optical readouts can provide a first-level indication of compositional changes that are subsequently resolved quantitatively using ATR-FTIR analysis. Such cross-modal agreement supports the feasibility of tiered sensing workflows in which colorimetric screening is complemented by vibrational spectroscopy for enhanced chemical discrimination in breath-relevant aerosol detection platforms [9,19,34].

### 3.6. Implications for Breath-Relevant Aerosol Detection and Multimodal Sensing

Exhaled breath aerosols contain complex mixtures of inorganic salts, proteins, metabolites, extracellular vesicles, and inflammatory mediators whose relative abundances reflect airway physiology and disease-associated biochemical processes. The ability of MCPR films to capture both ionic and proteinaceous aerosol components while preserving chemically interpretable optical and vibrational signatures demonstrates their suitability as integrated substrates for multimodal breath-aerosol sensing platforms [2,5,6,48]. Furthermore, the observed dependence of ATR-FTIR spectral responses on environmental pH highlights the influence of airway surface liquid chemistry on aerosol composition and suggests that local microenvironmental acidity may modulate detectable biomarker signatures in breath-derived samples. Because airway surface liquid pH is known to vary across respiratory conditions, including infection, inflammation, and obstructive lung disease, the coupled optical–vibrational responsiveness observed in MCPR films provides a useful framework for interpreting compositional variability in physiologically relevant aerosol microenvironments [7,49,50].

In particular, the strong correlation observed between λmax shifts and sulfate-to-protein spectral ratios suggests that MCPR films enable simultaneous monitoring of ionic balance and protein-associated aerosol signatures within a single sensing interface. This cross-modal agreement supports the feasibility of tiered sensing workflows in which rapid colorimetric screening is complemented by quantitative vibrational spectroscopy for enhanced compositional discrimination of breath-derived particulate samples. Together, these findings indicate that MCPR-based sensing substrates represent promising low-cost capture-and-readout platforms for future breath-analysis strategies targeting respiratory infection screening, inflammatory airway disease monitoring, and environmental exposure assessment. The current workflow should be interpreted as a proof-of-concept capture-and-readout platform rather than a real-time diagnostic device. The colorimetric response of MCPR films may provide a rapid first-level visual or optical indication, while ATR-FTIR analysis provides confirmatory compositional information. Future development will focus on reducing aerosol collection time, integrating portable spectroscopic hardware, and automating spectral analysis to improve practical deployment. Because the present study was designed as a proof-of-concept validation using fixed model aerosol formulations, formal LOD and LOQ values were not determined. The current work establishes the feasibility of aerosol capture, optical responsiveness, and ratiometric ATR-FTIR discrimination rather than trace-level biomarker quantification. Future analytical validation will require concentration-dependent calibration studies to define LOD, LOQ, linear dynamic range, and matrix-interference effects using both model aerosols and authentic exhaled breath samples. Water, chloride, phosphate, carbonate, lipids, mucins, metabolites, and changing aerosol loading are all possible interferents in authentic breath aerosols. These components may have an impact on film hydration, optical response, FTIR band overlap, and AUC-based ratio measures. Future research should assess selectivity and matrix interference utilizing controlled mixed-component panels and actual exhaled breath aerosol samples.

## 4. Conclusions

MCPR films function as integrated multimodal substrates that capture aerosolized biomolecular components while preserving chemically interpretable optical and vibrational signatures. Using model aerosol systems representing ionic and proteinaceous constituents relevant to exhaled breath particles, we showed that MCPR films enable quantitative discrimination between sulfate and protein contributions through ratiometric ATR-FTIR analysis across physiologically relevant pH conditions. Complementary UV-Vis measurements confirmed reproducible indicator-based optical transitions that closely correlated with FTIR-derived compositional metrics, supporting the presence of a shared pH-responsive sensing microenvironment within the polymer matrix. Morphological and elemental characterization performed on methylcellulose capture films further verified efficient deposition of particulate, ionic, and proteinaceous aerosol components, establishing the suitability of the underlying cellulose matrix as a stable aerosol capture interface for downstream spectroscopic interrogation.

These findings indicate that MCPR films integrate aerosol capture with coupled optical and vibrational sensing responses, providing a practical framework for resolving compositional variability in mixed aerosol deposits using a single low-cost sensing substrate. The observed agreement between colorimetric λmax shifts and sulfate-to-protein FTIR ratio metrics further supports the feasibility of tiered sensing workflows in which rapid visual screening can be complemented by quantitative vibrational spectroscopy. Future work should focus on evaluating detection limits for clinically relevant breath biomarkers, validating performance using authentic exhaled breath aerosol samples, and integrating automated spectral interpretation approaches to support portable and field-deployable breath-analysis platforms for respiratory monitoring and exposure assessment.

## Data Availability

The original contributions presented in this study are included in the article/Supplementary Material. Further inquiries can be directed to the corresponding author.

## Supplementary Materials

The following supporting information can be downloaded at: https://www.mdpi.com/article/10.3390/s26123839/s1, Figure S1: Reference ATR-FTIR spectrum of methylcellulose (MC) control film prior to aerosol exposure.
