# Supplementary material for: Multimodal Optical and Ratiometric ATR-FTIR Discrimination of Mixed Aerosol Components Using pH-Responsive Methylcellulose–Phenol Red Films
Source: Sensors (Basel). 2026 Jun 17;26(12):3839. doi: 10.3390/s26123839 (PMC13307151; doi:10.3390/s26123839)
Supplement: Supplementary file 1 [file sensors-26-03839-s001.zip › sensors-4301397-supplementary.pdf]

Supplementary Information

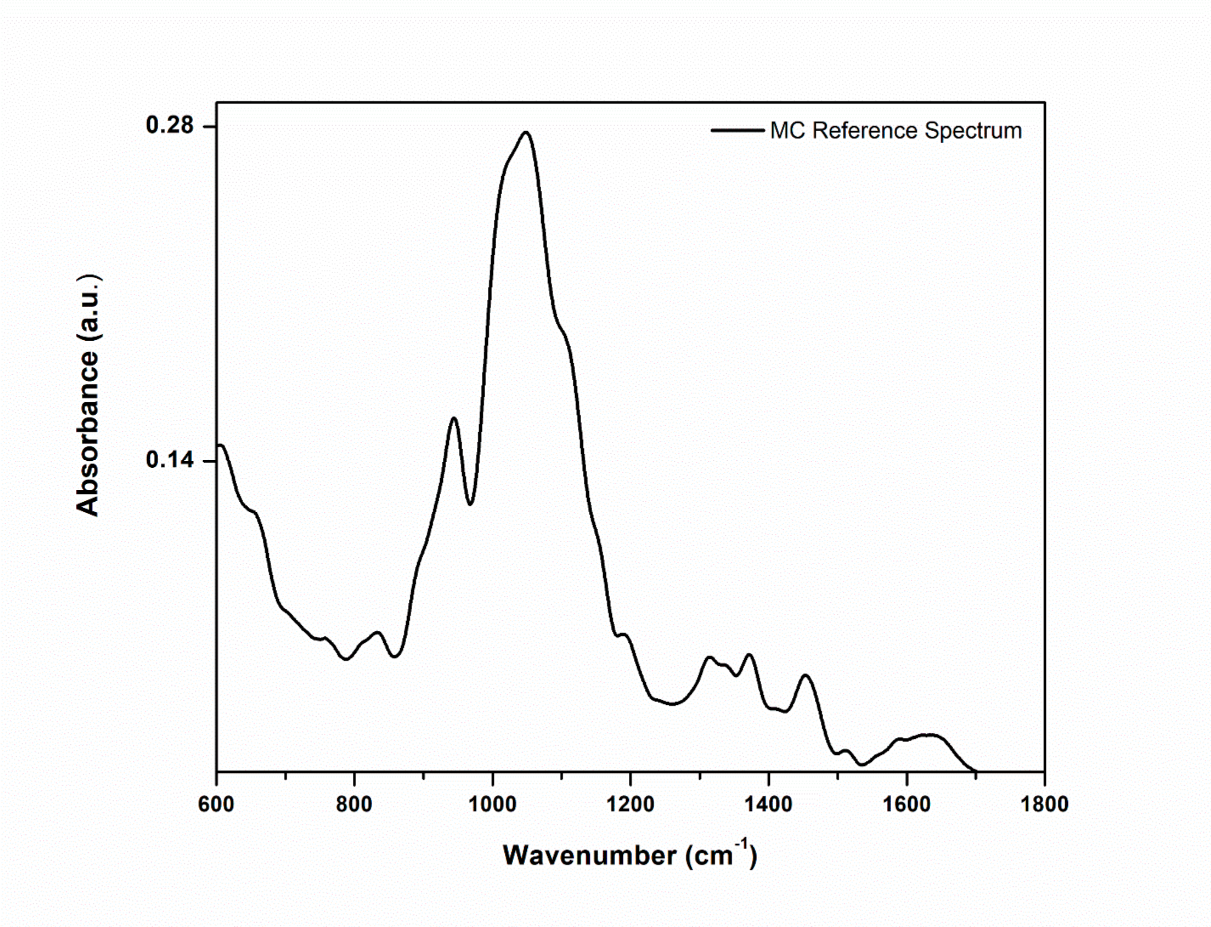

Figure S1. Reference ATR-FTIR spectrum of methylcellulose (MC) control film prior to aerosol exposure. The unexposed MC reference spectrum shows the baseline polymer-associated absorbance features used to distinguish film-background contributions from aerosol-derived sulfate and protein signals in exposed samples.
